# Pulmonary Atresia with Intact Ventricular Septum associated with Ventriculo-Coronary Arterial Communication in a Fetus at 21 Weeks of Gestation

**DOI:** 10.1155/2020/7581483

**Published:** 2020-07-15

**Authors:** Can Yilmaz Yozgat, Yanki Celik Yilmazer, Yilmaz Yozgat

**Affiliations:** ^1^Faculty of Medicine, Bezmialem Vakif University, Istanbul, Turkey; ^2^LaMED Private Radiology Clinic, Izmir, Turkey; ^3^Department of Pediatric Cardiology, Bezmialem Vakif University, Istanbul, Turkey

## Abstract

Pulmonary atresia with the intact ventricular septum (PA-IVS) is a rare anomaly that has an absent communication between the right ventricle and pulmonary arterial circulation. PA-IVS has a hypoplastic and hypokinetic and thickened right ventricle with the intact ventricular septum. It can be diagnosed with PA-IVS in routine obstetric ultrasound examination because the four-chamber view of PA-IVS is generally abnormal. The size of the right ventricular cavity is proportional to the *Z* value of the diameter of the tricuspid valve. The essential associated cardiac finding of PA-IVS is ventricular-coronary arterial communications (VCAC). The hypertensive RV forces blood through the intramyocardial sinusoids with continuous fistulous contact with the epicardial branches of RCA. It is called the VCAC. A color Doppler can detect VCAC due to its location in the pericardium along the coronary artery. If patients have VCAC and tricuspid *Z* score below -4, all of these conditions are infallible signs of high mortality rates in their fetal or postnatal lives. Our aim in presenting this case is to remind perinatologists if they detect an abnormal four-chamber view of the fetus's heart, they should also carefully examine whether VCAC exists. Herein, we report a case of PA-IVS and VCAC at 21 weeks' gestation with fetal echocardiographic images.

## 1. Introduction

Pulmonary atresia with the intact ventricular septum (PA-IVS) is a congenital cardiac anomaly which occurs due to the disjoint communication between the right ventricle (RV) and the pulmonary arterial circulation. The circulation is associated with an intact ventricular septum, which does not allow any connections between the RV and the left ventricle (LV). PA-IVS has a prevalence rate of 0.042-0.053 per 1000 live births [1]. PA-IVS has hypoplastic and hypokinetic and thickened right ventricle with the intact ventricular septum. It can be diagnosed with PA-IVS in routine obstetric ultrasound examination because the four-chamber view of PA-IVS is generally abnormal [2, 3]. The size of the heart is usually is normal. The most integral cardiac association of PA-IVS is ventricular-coronary arterial communication (VCAC), which is coronary artery abnormality. It is approximately one-third of the patient with PA-IVS. A color Doppler can detect VCAC due to its location in the pericardium along the coronary artery. Herein, we report a case of pulmonary atresia with intact ventricular septum and VCAC at 21 weeks' gestation with fetal echocardiographic images.

## 2. Case Report

**Figure 1:**
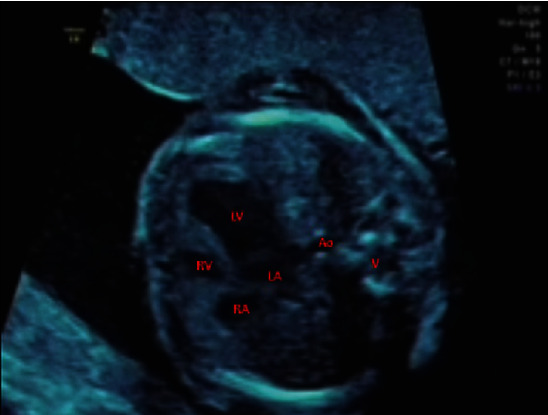
Apical four-chamber view showing the right ventricular cavity was moderately hypoplastic with the hypertrophic myocardium and intact ventricular septum.

**Figure 2:**
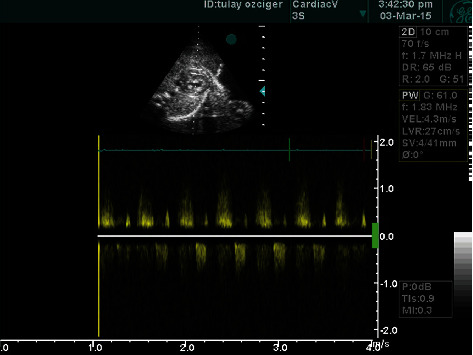
Pulsed Doppler examination of VCAC demonstrating bidirectional flow.

A female, 32-year-old gravida 2, para 2 who had an initial diagnosis of abnormal four-chamber view of the fetus's heart, was referred to our clinic due to its reputation of advanced research and management. Her first son was healthy. She did not have any systemic illness or a history of chromosomal anomaly, and she had not taken any medication, alcohol, or cigarettes before her admission to our clinic. Her routine obstetric sonographic examination was unremarkable as well. Fetal echocardiography was performed. On fetal echocardiography, a small right ventricle, hypertrophic myocardium, and intact ventricular septum were observed in the four-chamber view of the heart (Figure 1). The tricuspid valve annulus was 3.7 mm (*Z* score -4.75), and the pulmonary valve annulus was 2.8 mm (*Z* score -3.77). The pulmonary artery had an extremely thick pulmonary valve. Antegrade pulmonary blood flow to the lungs was not detected (thick membranous pulmonary atresia), and the pulmonary arteries were perfused by retrograde ductus arteriosus flow. Another intriguing finding of the color Doppler examination was that it detected the turbulent flow from the apex along the pericardium via the right coronary artery to the aorta. This condition is known as VCAC (video 1). Pulse waved Doppler interrogation was performed to demonstrate and further prove the findings of VCAC bidirectional flow (systolic flow towards the aorta and diastolic flow towards the right ventricle cavity) (Figure 2). Our pediatric cardiology department made the diagnosis of PA-IVS and VCAC. Prenatal workup revealed no signs of other fetal anomalies, and the karyotype obtained following amniocentesis was normal. We consulted with the Department of Maternal-Fetal Medicine to determine the possible treatment options for the patient. In the end, our team decided that there was not any right way to convalesce the patient's predicament. Our council decided that the best way to go forward was to terminate the pregnancy. The patient also accepted this decision.

## 3. Discussion

Throughout the fetal life, the prognosis of PA-IVS is subject to change, and the prognosis will depend upon the size of RV's cavity and its functions. There is a strong positive association between having a good prognosis in fetal life and having an excellent biventricular hole. If the RV inlet and outlet septum are well-preserved, pulmonary valve perforation will be performed in the fetal or neonatal life [3, 4]. Patients with PA-IVS have extremely high RV pressures in both systole and diastolic phases. The hypertensive RV forces blood through the intramyocardial sinusoids with continuous fistulous communication with the epicardial branches of RCA. During the systolic period of the heart, the central outflow of the hypertensive RV is from the RV to the aorta (retrograde filling). However, this situation is vice versa in the diastolic phase [1, 5].

The retrograde filling of the coronary circulation, which flows from the hypertensive RV, is known as the RV-dependent coronary circulation (RVDCC). Patients with RVDCC can also have atresia or stenosis in their proximal major coronary arteries. This deteriorated condition can be visualized by an angiographic study in postnatal life [5]. Even though our case had RVDCC, we were not able to detect whether the case, at the same time, had atresia or stenosis in her proximal major coronary arteries. If patients have tricuspid valve regurgitation, VCAC, and tricuspid *Z* score below -4, all of these conditions are infallible signs of high mortality rates in their fetal or postnatal lives. Death in utero may occur, with overall mortality in live-borne children of greater than 50% [6]. It is not recommended to perform surgical RV decompression (RV to pulmonary artery valve conduit/pulmonary valvuloplasty) in the postnatal life to treat the patients with RVDCC because it might also instigate catastrophic myocardial infarction or even myocardial rupture.

Single-ventricle palliation is often the best treatment option for RVDCC. RVDCC accounts for almost all of the mortality in patients with PA-IVS [5] in the postnatal life. Hence, diagnosing VCAC in utero is vital for family counseling and possible to optimize prenatal care as well. Our case had a ventricular corner artery communication and a tricuspid *Z* score below -4, which was indicative of a bad prognosis. This case was consulted with the Department of Maternal-Fetal Medicine, and our team decided to terminate the patient's pregnancy.

## Supplementary Materials

Supplementary Materials The color Doppler examination demonstrating the turbulent flow from the apex along the pericardium via the right coronary artery to the aorta (VCAC).
